# Supplementary material for: mTORC2 interactome and localization determine aggressiveness of high-grade glioma cells through association with gelsolin
Source: Sci Rep. 2023 Apr 29;13:7037. doi: 10.1038/s41598-023-33872-y (PMC10148843; doi:10.1038/s41598-023-33872-y)
Supplement: Supplementary file 1 — Supplementary Information 1. [file 41598_2023_33872_MOESM1_ESM.docx]

**Supplementary Information**

**mTORC2 Interactome and Localization Determine Aggressiveness of High-Grade Glioma Cells through Association with Gelsolin**

Naphat Chantaravisoot^1,2†*^, Piriya Wongkongkathep^2,3†^, Nuttiya Kalpongnukul^2^, Narawit Pacharakullanon^1^, Pornchai Kaewsapsak^1,4^, Chaiyaboot Ariyachet^1,5^, Joseph A. Loo^6,7,8^, Fuyuhiko Tamanoi^9,10^ and Trairak Pisitkun^2,3*^

^†^Equal first authors

^*^Corresponding authors: Naphat Chantaravisoot (naphat.c@chula.ac.th) and Trairak Pisitkun (trairak@gmail.com)

^1^ Department of Biochemistry, Faculty of Medicine, Chulalongkorn University, Bangkok, 10330, Thailand.

^2^ Center of Excellence in Systems Biology, Faculty of Medicine, Chulalongkorn University, Bangkok, 10330, Thailand.

^3^ Research Affairs, Faculty of Medicine, Chulalongkorn University, Bangkok, 10330, Thailand.

^4^ Research Unit of Systems Microbiology, Faculty of Medicine, Chulalongkorn University, Bangkok, 10330, Thailand.

^5^ Center of Excellence in Hepatitis and Liver Cancer, Faculty of Medicine, Chulalongkorn University, Bangkok, 10330, Thailand.

^6^ Department of Chemistry and Biochemistry, University of California, Los Angeles, CA, 90095, USA.

^7^ UCLA/DOE Institute of Genomics and Proteomics, University of California, Los Angeles, CA, 90095, USA

^8^ Department of Biological Chemistry, University of California, Los Angeles, CA, 90095, USA..

^9^ Department of Microbiology, Immunology & Molecular Genetics, University of California, Los Angeles, CA, 90095, USA.

^10^ Institute for Integrated Cell-Material Sciences, Institute for Advanced Study, Kyoto University, Kyoto, 606-8501, Japan.

Send correspondence to:

Naphat Chantaravisoot

Dept. of Biochemistry, Faculty of Medicine

Chulalongkorn University

1873 Rama IV Pathumwan Bangkok, 10330, Thailand.

Email: naphat.c@chula.ac.th

ORCID ID: 0000-0003-3946-1798

Tel: +662-256-4482

**Contents**

**Supplementary Tables**

**Supplementary Table 1** The proteins proximally associated with RICTOR in U87MG cells. (Supplementary Table 1.xlsx)

**Supplementary Table 2** mTORC2 Interactome in U87MG cells under starvation condition. (Supplementary Table 2.xlsx)

**Supplementary Table 3** mTORC2 Interactome in U87MG cells under serum activation **condition.** (Supplementary Table 3.xlsx)

**Supplementary Table 4** mTORC2 Interactome in U87MG cells under mTOR inhibition condition by AZD8055. (Supplementary Table 4.xlsx)

**Supplementary Table 5** Differential expression of common mTORC2 Interactome in U87MG cells after mTORC2 activation by serum. (Supplementary Table 5.xlsx)

**Supplementary Table 6** Differential expression of common mTORC2 Interactome in U87MG cells after mTOR inhibition by AZD8055. (Supplementary Table 6.xlsx)

**Supplementary Table 7** Unique mTORC2 interactome found in serum-activated U87MG cells. (Supplementary Table 7.xlsx)

**Supplementary Table 8** Dynamic mTORC2 interactome inversely regulated in mTORC2 activation vs. inhibition by AZD8055 treatment. (Supplementary Table 8.xlsx)

**Supplementary Table 9** Differential expression of common proteins in U87MG compared to H4 cells. (Supplementary Table 9.xlsx)

**Supplementary Table 10** Differential expression of common mTORC2 interactome in U87MG compared to H4 cells. (Supplementary Table 10.xlsx)

**Supplementary Table 11** Differential expression of common mTORC2 interactome in wild-type compared to GSN knockdown U87MG cells. (Supplementary Table 11.xlsx)

**Supplementary Table 12** Final list of mTORC2 interactome commonly found in the samples from every protein digestion method. (Supplementary Table 12.xlsx)

**Supplementary Videos**

**Supplementary Video 1** Directional migration (16 hr) of normal and 2.0 µM AZD8055-treated U87MG cells.

**Supplementary Video 2** Cell migration recovery by serum supplementation (18 hr) of siRICTOR-treated cells.

**Supplementary Video 3** Live-cell imaging of U87MG cells containing actin labeled with SiR-Actin under normal and AZD8055-treated conditions.

**Supplementary Video 4** Live-cell imaging experiment showing microtubule networks in U87MG cells under serum activation, rapamycin (100 nM, 24 hr), AZD8055 (2.0 µM, 24 hr).

**Supplementary Video 5** Live-cell imaging experiment showing microtubule networks in U87MG cells under activated and *RICTOR* knockdown conditions.

**Supplementary Figures**

**Supplementary Fig. 1** Snapshot of U87MG live-cell imaging under different treatment, related to Fig. 1, Supplementary video 4 and 5.

Snapshot from live-cell imaging experiments showing actin cytoskeleton and microtubule networks in U87MG cells under serum activation, rapamycin (100 nM, 24 hr), AZD8055 (2.0 µM, 24 hr), and *RICTOR* knockdown. Cells were labeled with SiR-Actin and SiR-Tubulin. Scale bar, 20 µm.


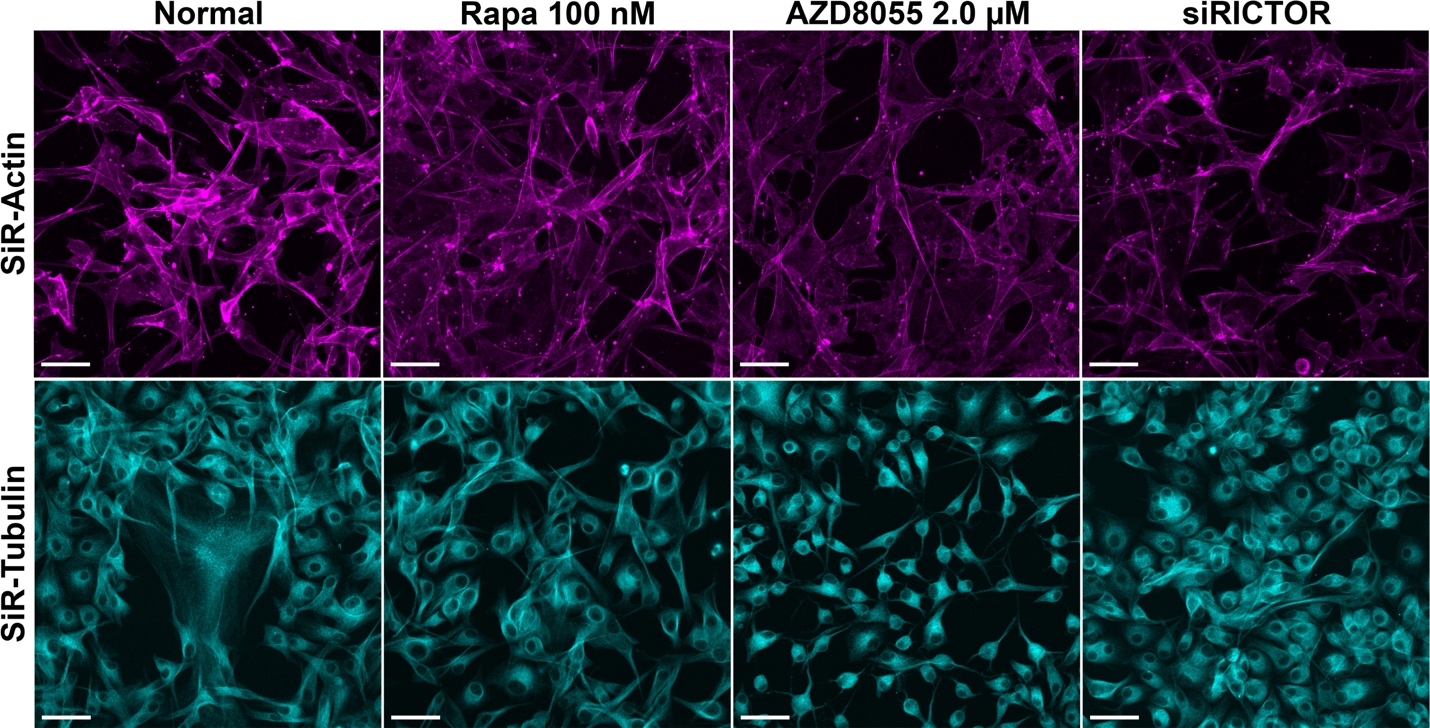


**Supplementary Fig. 2** Comparisons of high- vs. low-grade glioma cells, related to Fig. 5.

**(A)** Proliferation assays of high-grade (U87MG) and low-grade glioma (H4) cells. Cells were treated under two conditions: serum activation and inhibition. Cells were collected followed by MTS assays at 24, 48, and 72 hr. Each experiment was performed in three biological replicates containing technical triplicates. Statistical significance was calculated at the using Ordinary one-way ANOVA with Tukey’s multiple comparisons test.

**(B)** Wound-healing migration assay of low-grade glioma (H4) cells for 16 hours. Scale bar, 200 µm.

**(C)** Venn diagram showing the number of identified proteins from the cell lysate of U87MG and H4 cells. Cytoskeleton-associated proteins specifically identified in each cell line are listed (red: MF-associated proteins, blue: IF-associated proteins, green: MT-associated proteins).

**(D)** Heatmap showing Log_2_ LFQ of commonly found cytoskeletal proteins in U87MG and H4 cell lysates from the whole-cell lysate proteomics data; n = 3. Red boxes indicate mTORC2 interactome previously identified from U87MG cells.

**(E)** Immunofluorescence staining of RICTOR and VIM in U87MG and H4 cells. Scale bar = 20 µm.

**(F)** Venn diagram showing mTORC2 interactome identified from U87MG and H4 cells by immunoprecipitation using anti-RICTOR antibody.

**(G)** Heatmap showing Log_2_ LFQ of commonly found cytoskeletal proteins identified from the AP-MS data using anti-RICTOR antibody


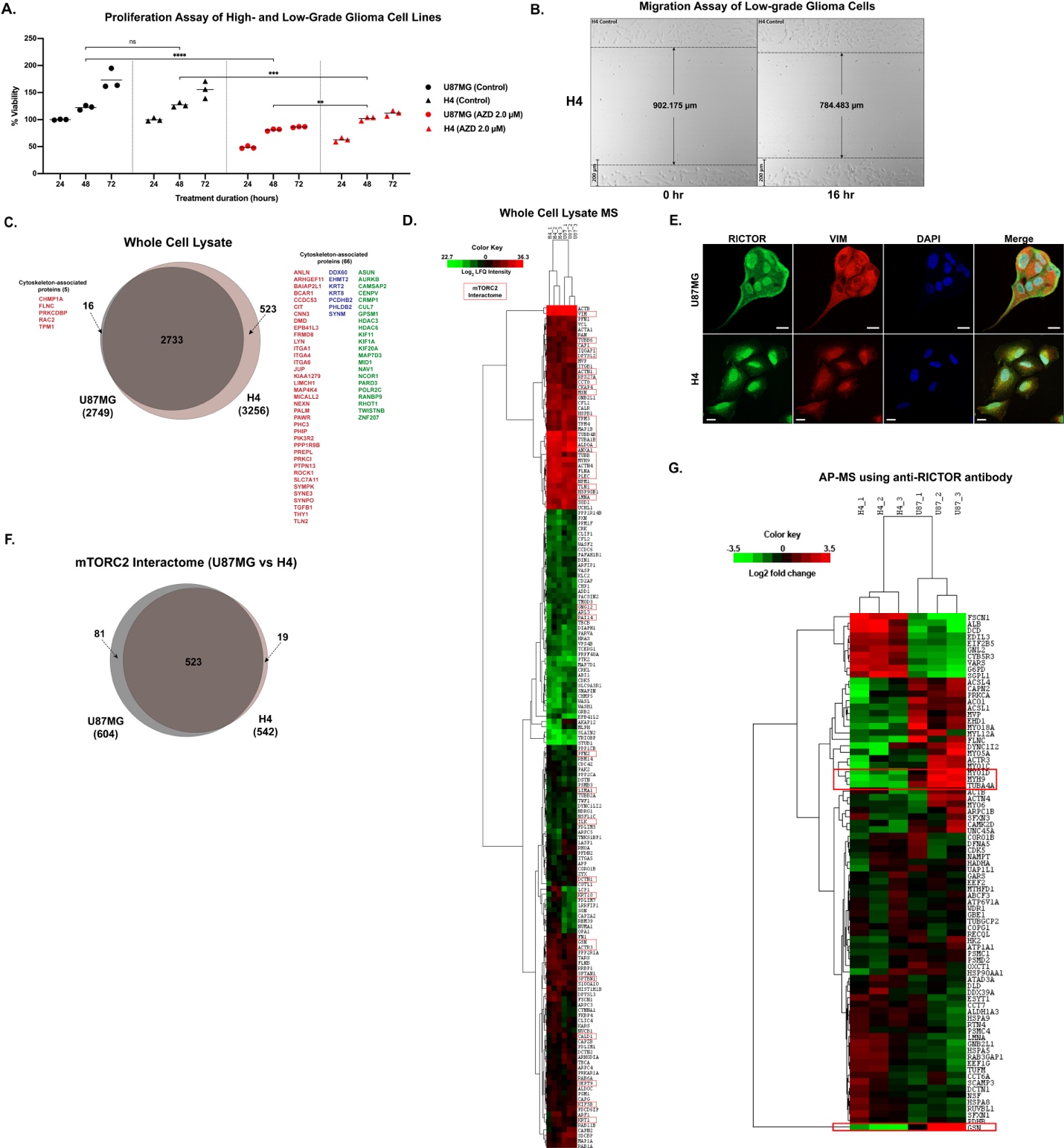


**Supplementary Fig. 3** Cell migration ability and mTORC2-GSN association in high- vs. low-grade glioma cells, related to Fig. 5.

**(A)** Wound-healing migration assays of high-grade glioma (U87MG and DBTRG-05MG) cells and low-grade glioma (H4 and SW1088) cells. Pictures were taken at 0, 6, 12 and 24 hours.

**(B)** Western blot analysis of immunoprecipitated RICTOR samples and whole-cell lysate (WCL) of four glioma cell lines including U87MG (U87), DBTRG-05MG (DBTRG), H4, and SW1088 under normal condition.

**
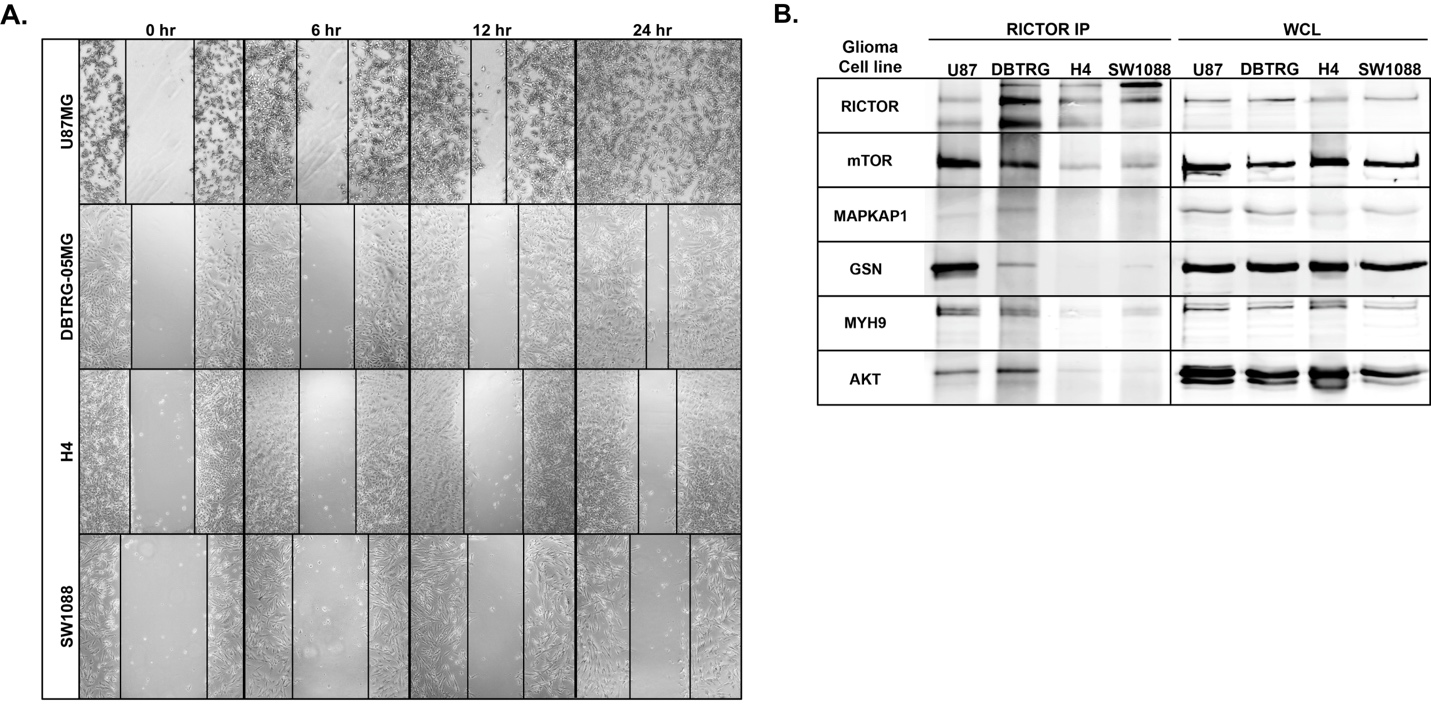
**

**Supplementary Fig. 4** Analysis of GSN and MYH9 as crucial interacting partners of mTORC2, related to Fig. 6.

**(A)** Immunofluorescence staining of U87MG cells showing the localizations of RICTOR and GSN under serum activation and inhibition (2.0 µM AZD8055, 2.0 µM JR-ABR-011, and 100 nM rapamycin) conditions. Scale bar = 20 µM.

**(B)** Immunofluorescence staining of U87MG cells showing the localizations of RICTOR and MYH9 under serum activation and inhibition (serum starvation, 2.0 µM AZD8055, 2.0 µM JR-ABR-011, and 100 nM rapamycin) conditions. Scale bar = 20 µM.

**(C)** Western blot analysis of RICTOR and GSN showing the protein levels after *GSN* knockdown

**(D)** Proteins in mTORC2 interactome identified from U87MG cells under normal condition compared to *GSN* knockdown.

**(E)** Ten top-ranked GO biological processes of mTORC2 interactome negatively affected after *GSN* knockdown.

**
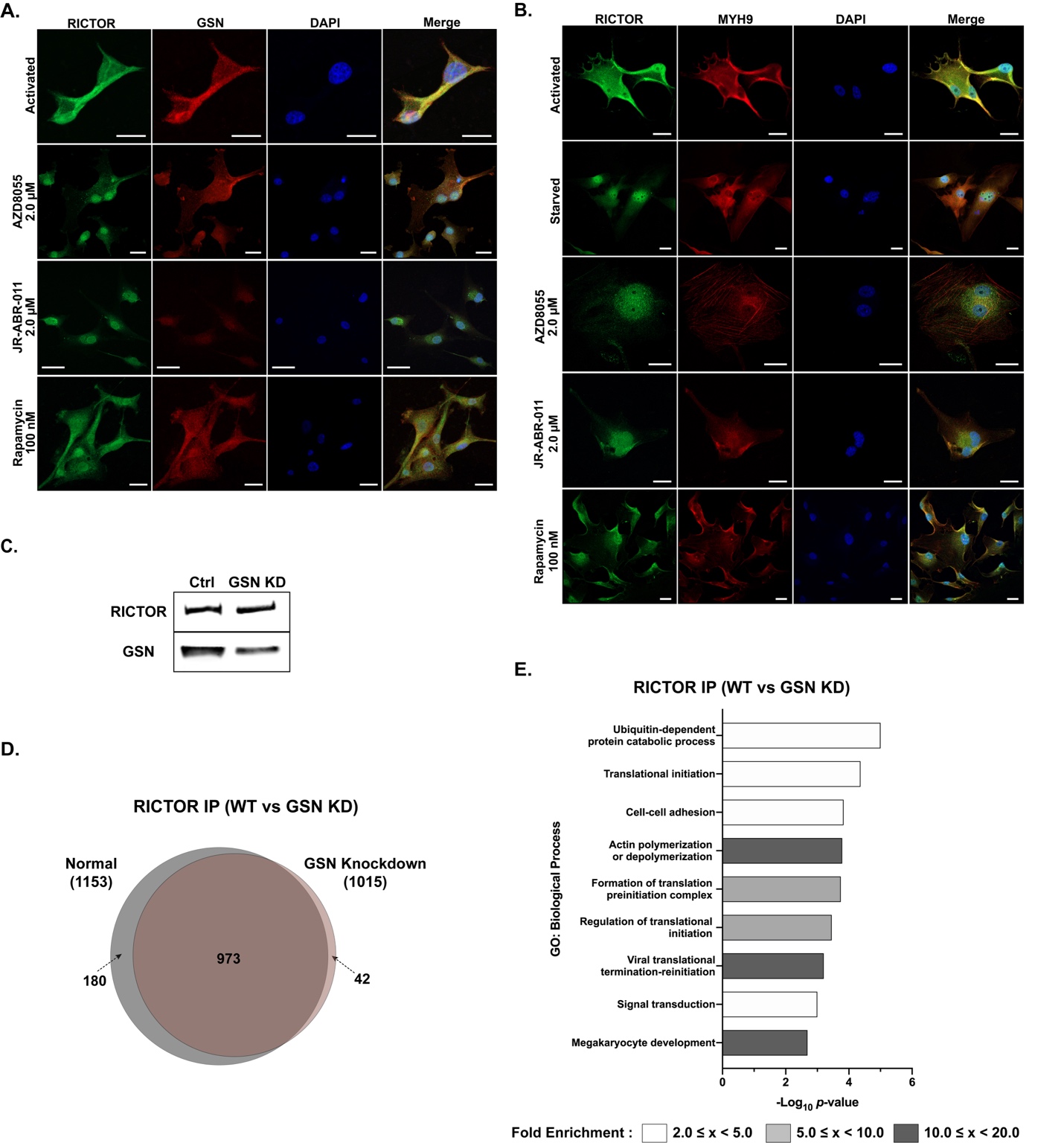
**

**Supplementary Fig. 5** mTORC2-GSN association in U87MG cells under different culturing conditions, and effects of *GSN* knockdown on cell migration ability, related to Fig. 6.

**(A)** Western blot analysis of immunoprecipitated RICTOR samples and whole-cell lysate (WCL) of U87MG cells under normal, serum activation, and inhibition (2.0 µM AZD8055 and 2.0 µM JR-ABR-011) conditions, showing the association between mTORC2 and its interactome.

**(B)** Wound-healing migration assays of U87MG cells under scrambled siRNA (scRNA) control vs. *GSN* knockdown conditions (siGSN). Pictures were taken at 0, 6, 12 and 24 hours.

**
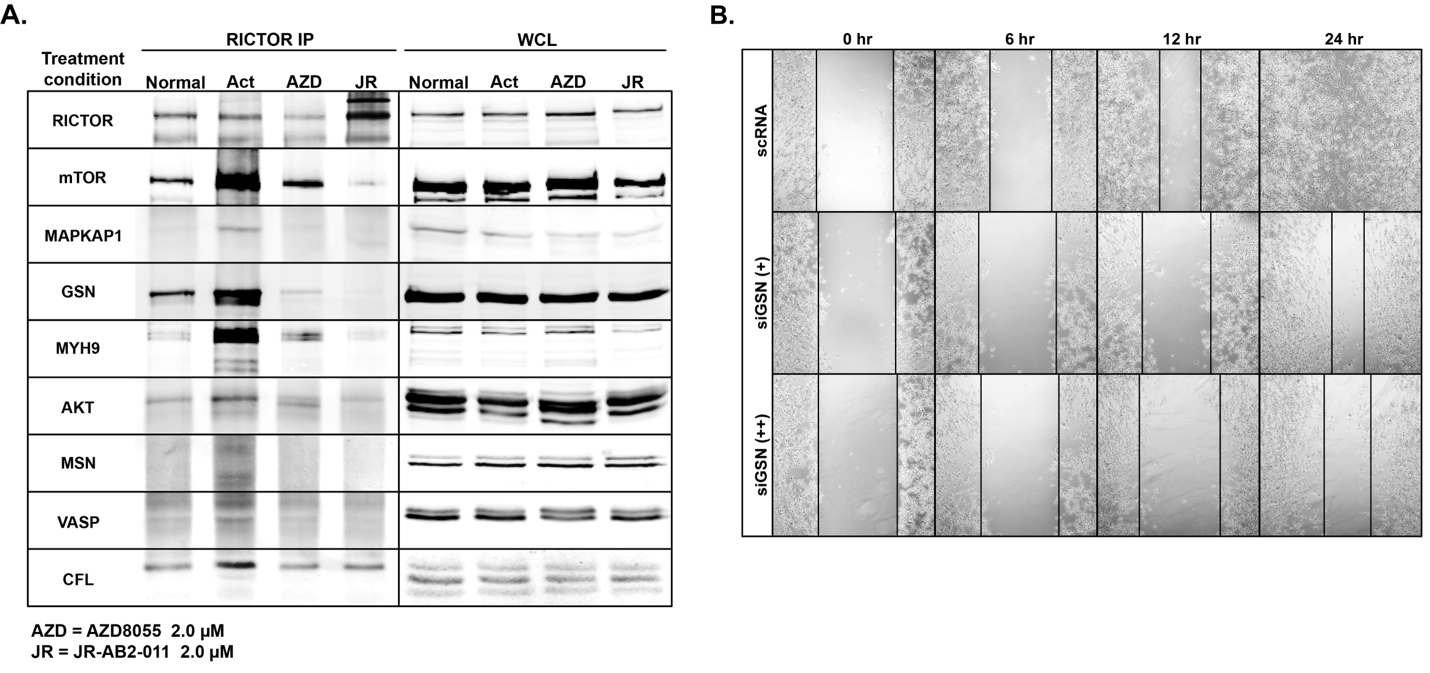
**

**Supplementary Fig. 6** mTORC2 Interactome Acquired by Different Proteomic Methods, Related to Fig. 7.

Overlap of mTORC2 stable interactome identified from each proteomic digestion method.


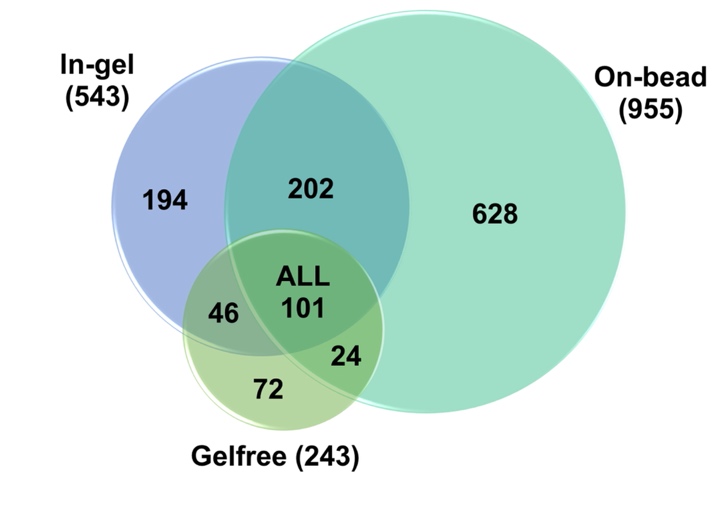


**Western blot membranes performed in the study**

**
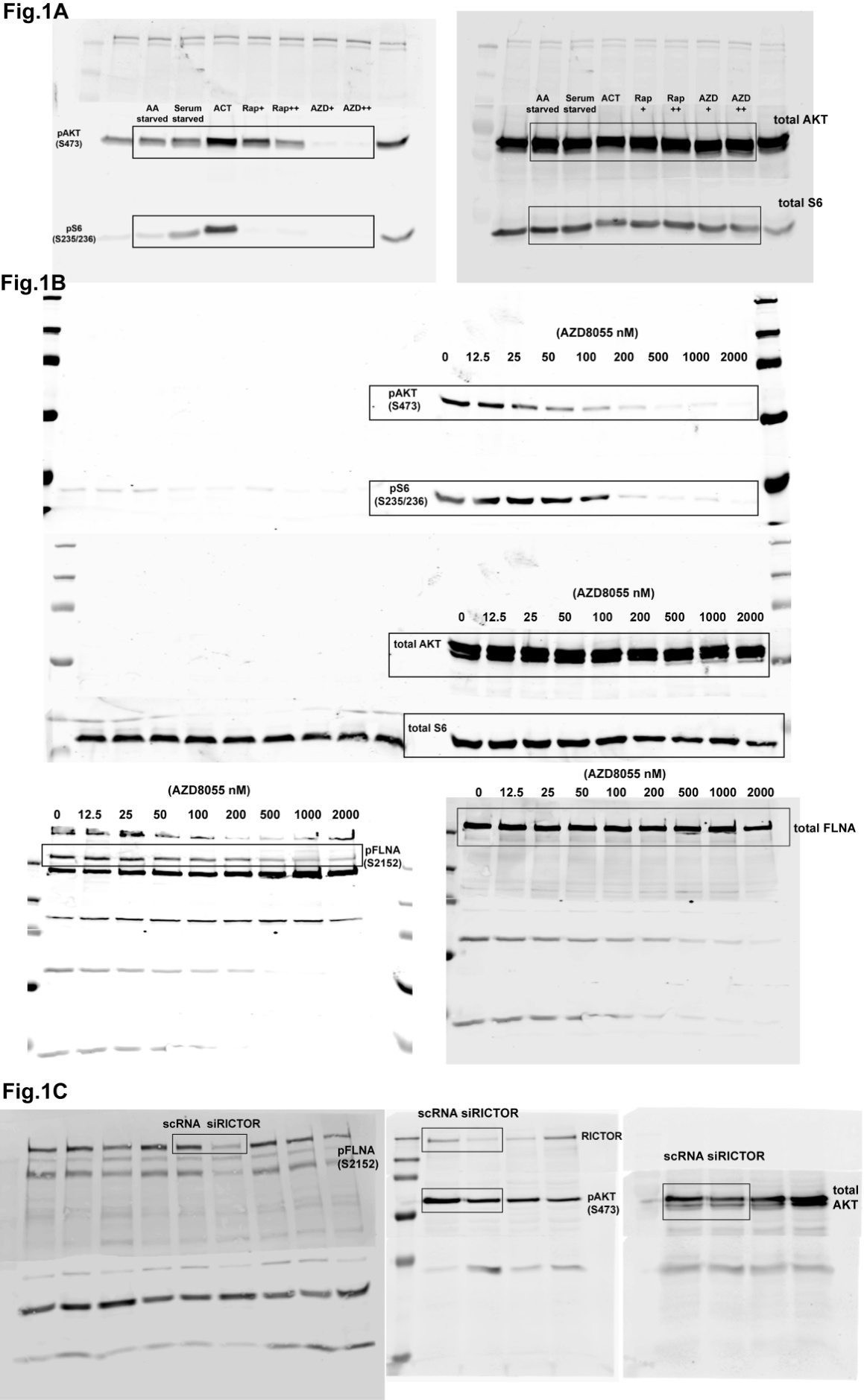
**

**
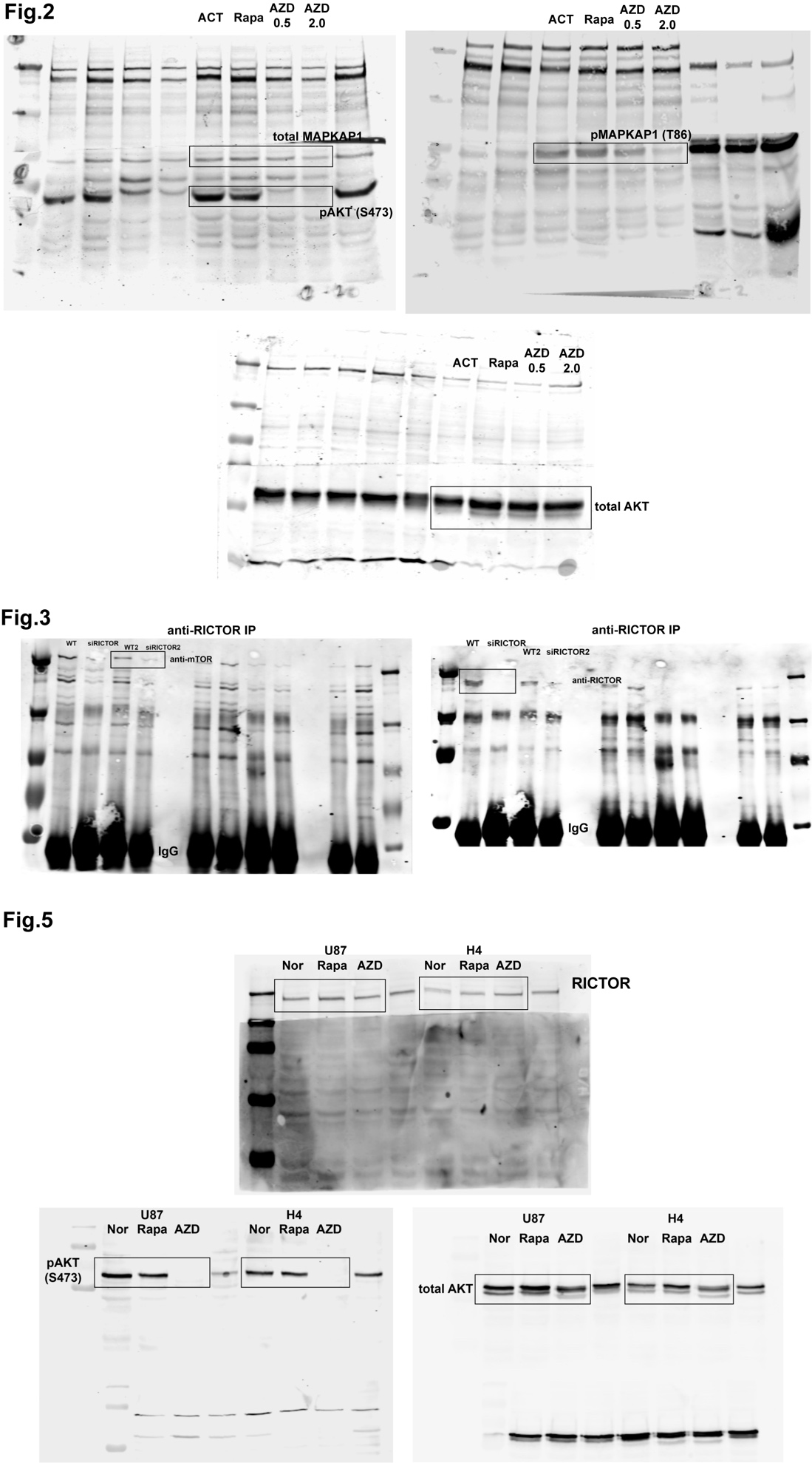
**

**
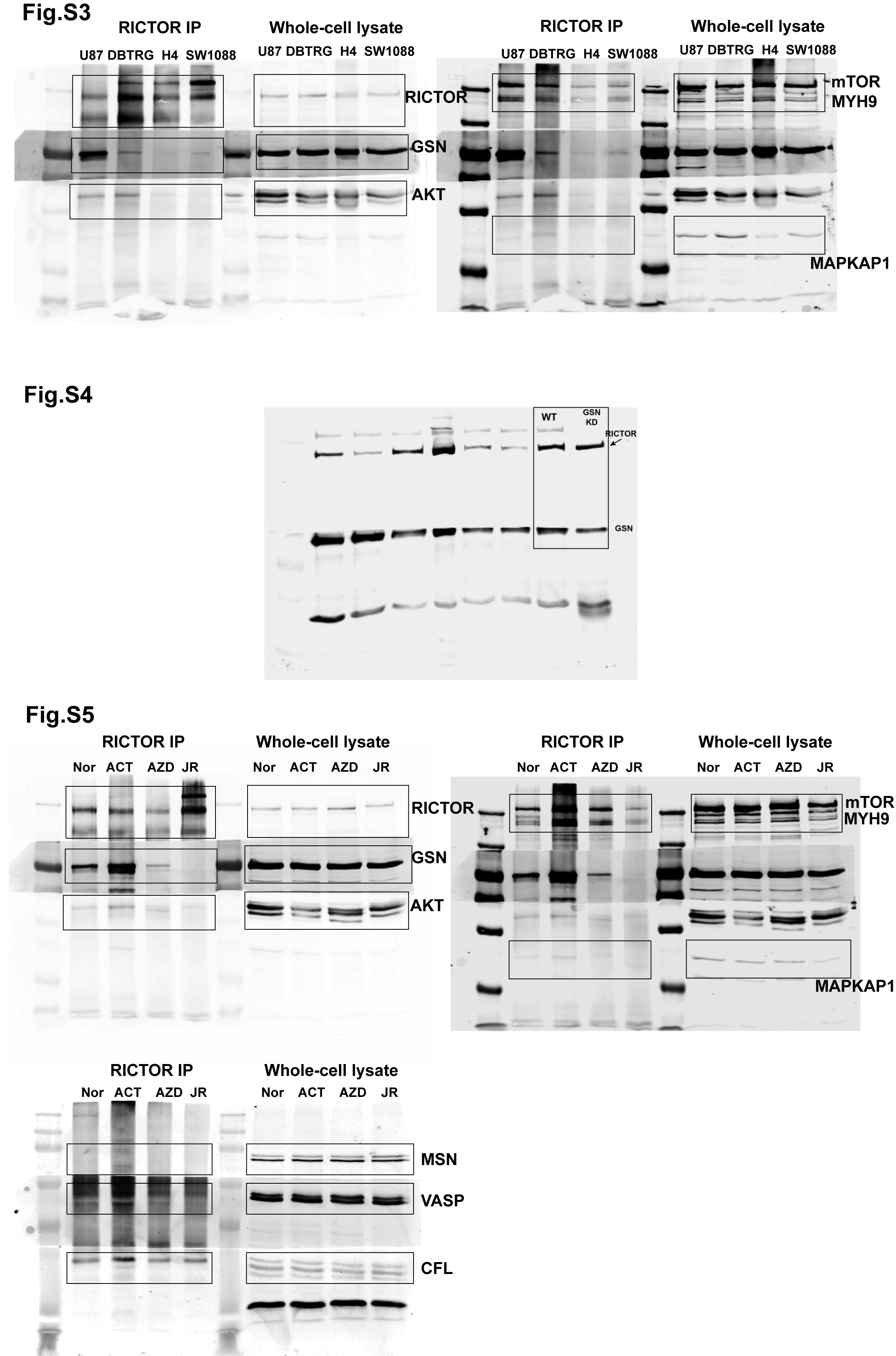
**
